# Gel-type autologous chondrocyte (Chondron™) implantation for treatment of articular cartilage defects of the knee

**DOI:** 10.1186/1471-2474-11-103

**Published:** 2010-05-28

**Authors:** Nam-Yong Choi, Byoung-Woo Kim, Woo-Jin Yeo, Haeng-Boo Kim, Dong-Sam Suh, Jin-Soo Kim, Yoon-Sik Kim, Yong-Ho Seo, Jea-Yeong Cho, Chung-Woo Chun, Hyun-Shin Park, Asode Ananthram Shetty, Seok-Jung Kim

**Affiliations:** 1 Department of Orthopedic Surgery St. Paul's Hospitial The Catholic University of Korea 620-56, Jeonnong 1-dong, Dongdaemun-gu, Seoul, 130-021 Korea; 2 The Department of Orthopedic Surgery The Leon Wiltse Memorial Hospital 994-3, Ingye-dong, Paldal-gu, Suwon-si, Gyeonggi-do, 442-070 Korea; 3 Department of Orthopedic Surgery Barunsesang Hospital 355-5, Yatap-dong, Bundang-gu, Seongnam-si, Gyeonggi-do, 463-828 Korea; 4 Haeng-Boo, Kim Orthopeadic Surgery , Myeongil-dong, Gangdong-gu, Seoul, 134-070 Korea; 5 RMS, SewonCellontech, 273-15, Seongsu 2-ga 3-dong, Seongdong-gu, Seoul, 133-831 Korea; 6 Department of Orthopedic Surgery Hyundae Hospital, 663, Janghyeon-ri, Jinjeop-eup, Namyangju-si, Gyeonggi-do, 472-865 Korea; 7 Department of Orthopedic Surgery, New Korea Hospital 1764, Janggi-dong, Gimpo-si, Gyeonggi-do, 415-060 Korea; 8 Department of Orthopedic Surgery, Changwon Jungang Hospital 5, Namyang-dong, Changwon-si, Gyeongsangnam-do, 641-802 Korea; 9 Center for Sport Medicine, JY Cho Orthgopedic Surgery Clinic 699, Tanbang-dong, Seo-gu, Daejeon, 302-223 Korea; 10 Department of Orthopedic Surgery Murup Hospital, Jungang-dong 3-ga, Masan-si, Gyeongsangnam-do, 631-716 Korea; 11 King's College Hospital, Denmark Hill, London SE5 9RS, UK; 12 Orthopedic Department, Uijeongbu St. Mary's Hospital, Kumoh-dong, Uijeongbu City, Gyeonggi-do, 480-717, Korea

## Abstract

**Background:**

Gel-type autologous chondrocyte (Chondron™) implantations have been used for several years without using periosteum or membrane. This study involves evaluations of the clinical results of Chondron™ at many clinical centers at various time points during the postoperative patient follow-up.

**Methods:**

Data from 98 patients with articular cartilage injury of the knee joint and who underwent Chondron™ implantation at ten Korean hospitals between January 2005 and November 2008, were included and were divided into two groups based on the patient follow-up period, i.e. 13~24-month follow-up and greater than 25-month follow-up. The telephone Knee Society Score obtained during telephone interviews with patients, was used as the evaluation tool.

**Results:**

On the tKSS-A (telephone Knee Society Score-A), the score improved from 43.52 ± 20.20 to 89.71 ± 13.69 (P < 0.05), and on the tKSS-B (telephone Knee Society Score-B), the score improved from 50.66 ± 20.05 to 89.38 ± 15.76 (P < 0.05). The total improvement was from 94.18 ± 31.43 to 179.10 ± 24.69 (P < 0.05).

**Conclusion:**

Gel-type autologous chondrocyte implantation for chondral knee defects appears to be a safe and effective method for both decreasing pain and improving knee function.

## Background

As articular cartilage has only limited ability to regenerate, many treatment modalities have been developed during the past several decades to treat symptomatic articular cartilage injuries [1]. Among these treatment modalities, autologous chondrocyte implantation (ACI) has become a standard technique used to repair symptomatic, full-thickness, chondral injuries [2-4].

The traditional ACI technique involves injection of cultured autologous cartilage cells into the prepared cartilage defect which is covered by a periosteal flap. The technique requires extensive surgical exposure in order for the sutures to be watertight as well as an additional incision for harvesting the periosteum. In addition, cell leakage, graft detachment, and graft hypertrophy are recognized as potential problems [5].

To solve the periosteum-associated problems, many biomaterials have also been used for a new generation of ACI techniques in which cells are combined with bioactive, resorbable biomaterials such as collagen membrance [6], hyaluronan polymer [7], and copolymers of polylactin and polyglactin [8]. Using this method, the necessity of a second incision to harvest tibial periosteum can be avoided and the surgery time can be shortened. ACI using a collagen matrix is currently used as a membrane on which chondrocytes are seeded and cultured for several days before the membrane is cut to the correct size and shape of the defect. Although this technique has advantages over conventional ACI due to its simplicity and the fact that it does not use periosteum, there are some potential disadvantages regarding cell loss and detachment of the membrane.

The technique using chondrocyte cell suspension cannot cover the total condyle so that it is watertight in an arthritic knee, and there is also a high risk of breakdown of the treated lesion and its subsequent progression to arthritis. In addition, the technique using collagen membrane has similar risks when treating a large lesion as those noted with the total condyle.

We have developed a new technique that reduces the surgical difficulties and facilitates the attachment and even distribution of chondrocytes in a defect. This method is based on the transplantation of in vitro cultured autologous chondrocytes mixed with fibrin glue into a cartilage defect.

For several years we have used gel-type autologous chondrocyte (Chondron™) implantation without using periosteum or membrane. Our current study involved evaluation of the clinical results of gel-type ACI (GACI) at many clinical centers and at various time points during the postoperative follow-up.

## Methods

Data from 98 patients who underwent gel-type ACI (GACI) at ten Korean hospitals between January 2005 and November 2008, were included and were divided into two groups according to the length of the follow-up period, i.e. 13~24-month follow-up and greater than 25-month follow-up.

Institutional review board (Catholic University of Korea) approval was obtained for this study. Patient informed consent was not required by the institutional review board.

Among the 98 study patients, there were 54 males (55.1%), and 44 females (44.9%). Patient age ranged from 17 to 65 years (mean age = 43.72 years, SD = 9.10). The average defect size was 5.23 ± 2.70 cm^2 ^(range: 1.00 ~ 14.50 cm^2^), i.e. less than 4 cm^2 ^in 29 patients (29.6%), 4.0~7.9 cm^2 ^in 57 patients (58.2%), and greater than 8.0 cm^2 ^in 12 patients (12.2%). The average follow-up period was 24.35 ± 8.35 months (range: 13 ~ 52 months) with a 13~24-month follow-up in 58 patients (59.2%) and greater than a 25-month follow-up in 40 patients (40.8%) (Table 1).

**Table 1:** Patient Description

Gender	Male	n (%)	54 (55.1%)
	Female	n (%)	44 (44.9%)
Age (years)	Mean ± std (Min ~ Max)		43.72 ± 9.10 (17 ~ 65)
	<30 years	n (%)	10 (10.2%)
	30~39 years	n (%)	12 (12.2%)
	40~49 years	n (%)	53 (54.1%)
	> = 50 years	n (%)	23 (23.5%)
Defect location	Femoral condyle	n (%)	83 (84.7%)
	Other	n (%)	15 (15.3%)
Defect Size (cm^2^)	Mean ± std (Min ~ Max)		5.23 ± 2.70 (1.00 ~ 14.50)
	< 4.0 cm^2^	n (%)	29 (29.6%)
	4.0~7.9 cm^2^	n (%)	57 (58.2%)
	>8.0 cm^2^	n (%)	12 (12.2%)
F/u period (Months)	Mean ± std (Min ~ Max)		24.35 ± 8.35 (12~ 52)
	13~24 Months	n (%)	58 (59.2%)
	25 Months~	n (%)	40 (40.8%)

### Evaluation of patient suitability for study participation (inclusion and exclusion criteria)

The indications for ACI were clinically significant, symptomatic, cartilaginous defects (Outerbrige III-IV) of the knee joint in patients with one or more cartilage defects not exceeding 15 cm^2 ^for a single defect or 20 cm^2 ^for multiple defects. The knee joint must be stable and without a severe deformity greater than 5 to 10 degrees of the valgus or varus.

The exclusion criteria included advanced osteoarthritis (Kellgren-Lawrence Grading Scale >2) and inflammatory arthritis with severe deformity exceeding the above range. Patellofemoral instability, drug abuse history, and psychological problems were also included as patient exclusion criteria.

### Surgical techniques

We used a two-stage surgical technique. After completing the clinical and radiological assessments, arthroscopy was performed in order to measure the positions, sizes, and depths of the chondral defects and to identify chondral defects and abnormalities involving the menisci and ligaments. Knee standing AP, lateral, and tangential radiographs were obtained to evaluate the knee joint deformity and the potential presence of osteoarthritis. In addition, 200-300 mg of cartilage from a non-weight-bearing portion of the knee, generally the medial or lateral superior ridge of the condyle or the intercondylar notch, was collected and sent to the GMP-certified, cell-culturing facility (SewonCellontech, Korea) for processing using the CE/MDD-certified, human-cell-processing kit (CRM kit™, SewonCellontech, Korea).

Autologous chondrocyte implantation was performed when 1.2 × 10^7 ^chondrocytes/vial had been cultured for 4 ~ 6 weeks after the initial surgery. Autologous chondrocytes were aseptically processed. The cell viabilities of all supplied products were greater than 80% prior to the final packaging.

**Figure 1:**
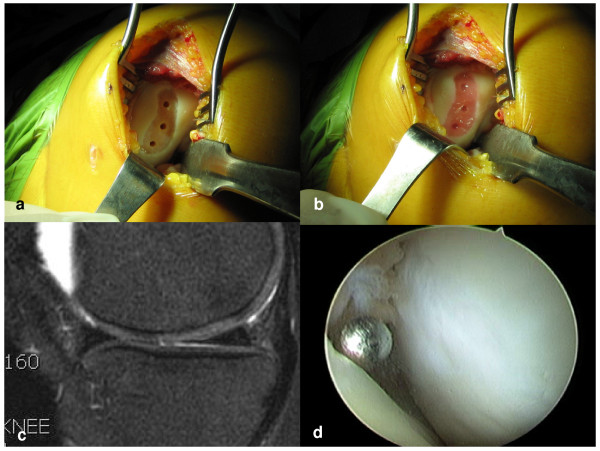
**The gel-type ACI (Chondron™) surgery**. a) Preparation of cartilage lesion, b) After injection of cell gel mixture, C) MRI one year postoperatively, D) Second-look arthroscopic view one year postoperatively.

During the second surgical procedure, arthrotomy was performed on the medial or lateral portion of the patella along the chondral defects. To ensure that the implanted chondrocytes merged well with normal cartilaginous tissue when exposed to defective areas, damaged cartilage was removed from the edges of the chondral defects. Multiple holes of 5-mm depth and 2.5-mm diameter were made at 1-2-cm intervals using a 2.5-mm drill bit so that the holes of the defect would receive the holding force of the graft (Fig. 1a). After release of the tourniquet, bone bleeding control was achieved using compression force applied to the holes using epinephrine-soaked gauze packing. For the injection procedure, two, 1-ml syringes and a Y-shaped mixing adapter were used. In one syringe, 1 ml of fibrinogen (Greenplast, Greencross, Korea) was filled with medium (CRM kit™, SewonCellontech, Korea), and the other syringe was filled with 0.9 ml of cell suspension and 0.1 ml of thrombin. Cultured autologous chondrocytes mixed with fibrin (1:1) were then slowly injected into the defect area (Fig. 1b).

In order not to overflow the margin, the dependent position of the defect site was maintained for five minutes. Flexion and extension motion of the knee was performed three to five times in order to check for any graft failure. The wound was then closed layer by layer. Use of continuous passive motion (CPM) machines was recommended for rehabilitation after surgery, followed by full weight bearing beginning 6~8 weeks postoperatively.

### Patient evaluations

**Figure 2:**
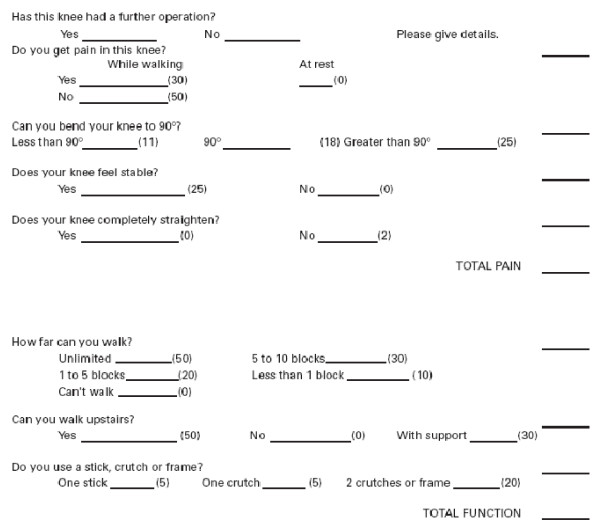
**The telephone score format used to estimate the KSS**.

Details concerning our patients' clinical outcomes were collected with the telephone score format initially used to estimate the KSS (Fig. 2)[9]. Recorded items included patient demographics, defect size, defect location, and the number of vials of chondrocyte used for implantation. The above information was obtained before implantation and again during telephone follow-up after post-implantation.

Our clinicians rated the patient knee status using the standardized 100-point rating scale of the telephone Knee Society Scoring system (tKSS) regarding pain (A) and function (B) (Fig. 2). Adverse event data were collected and included in the analysis regardless of whether or not they were considered to be related to the ACI.

### Statistical analysis

Categorical variables are presented as numbers and percentages, and continuous variables are expressed as mean ± SD and range. The improvements of tKSS-A and B scores were analyzed using either the paired *t *test or the Wilcoxon signed rank sum test, as indicated. To determine whether improvements were related to age, gender, defect size, defect location, the number of vials of chondrocyte used for implantation or the time interval between cartilage harvesting and ACI, we used the Mann-Whitney *U *test, Kruskal Wallis test, and Spearman's rho. All reported p-values were two-sided, and a p-value < 0.05 was considered statistically significant. SPSS software version 11.5 (SPSS Inc, USA) was used for statistical analyses.

### Cell and gel mixture ex-vivo evaluation

Fibrin-glue was used to develop the implantation protocol of gel-type cultured autologous chondrocytes. The order and ratio of mixing the major constituents of the fibrin glue with the cultured autologous chondrocytes, were optimized in order to guarantee maximal cellular viability and the ability to solidify within a practical length of time after implantation.

To measure the viability of chondrocytes mixed with fibrin according to the established protocol, cultured human chondrocytes (1.2 × 10^7^cells/vial) proliferated ex vivo, were collected. The collected cell suspension was mixed with the same volume of fibrin glue used to prepare 2 ml of cell-fibrin mixture gel. This gel was cultured for three days at 37°C in an 8% CO_2 _incubator. The viability of chondrocytes within the fibrin gel which was cultured for 0, 12, 24, 48, and 72 hours, was measured using MTT (3-(4,5-Dimethyl-2-thiazolyl)-2,5-diphenyl-2H-tetrazolium bromide, Sigma, USA.) assay.

In addition, the viable status of chondrocytes within the cell-fibrin matrix was grossly examined using Calcein-AM/Ethidium homodimer-1 (Invitrogen, USA) staining. Live cells were expressed as a green color and dead cells were expressed as a red color under fluorescent microscopy following the staining. A scanning electron microscope (Hitachi, Japan) was used to confirm the morphological structure of the mixture of chondrocytes and fibrin. The material safety of autologous chondrocytes mixed with fibrin was confirmed as lacking the toxicity seen in a subcutaneous toxicity study using C57BL/6 mice (Korea Institute of Toxicology, 2006).

## Results

**Figure 3:**
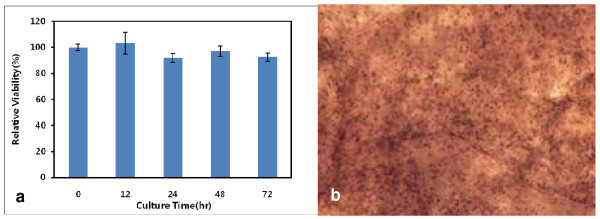
**The viability and distribution of chondrocytes within the cell-fibrin gel**. a) The relative cell viability profiles assayed by MTT assay. b) The distribution of chondrocytes within the gel.

**Figure 4:**
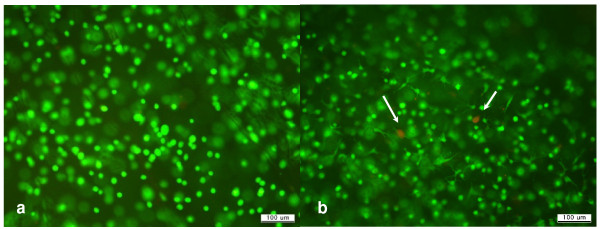
**The viable chondrocytes within the cell-gel mixture on Calcein-AM/Ethidium homodimer-1 staining**: a) A 0-hour and b) A 72-hour culture (×100). The white arrow in b) denotes the dead cells which appear as a reddish color.

**Figure 5:**
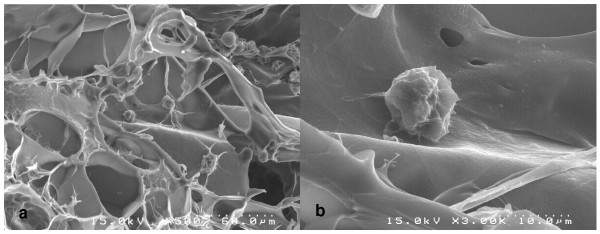
**A scanning electron microscopy picture showing the morphological structure of chondrocytes within the cell-fibrin gel**. The cell-fibrin gel was incubated in a CO_2 _incubator and was freeze-dried in order to reveal the scaffold structure. a) ×500, b) ×3000.

The MTT assay showed that the viability of the cells in the gel remained above 90% of the initial viability for 72 hours (Fig. 3a). The cells were found to be well mixed with fibrin and evenly distributed within the gel (Fig. 3b). The viable status and distribution of chondrocytes in the gel were also confirmed based on the Calcein-AM/Ethidium homodimer-1 staining. The viability of the cells in the cell-gel mixture, according to the fluorescence staining, was found to exceed 90% after 72 hours of culturing the mixture, as shown in the MTT assay (Fig. 4). We found that the pores were evenly present within the fibrin gel. We was also noted that the chondrocytes were evenly distributed and maintained a round shape within the pores on the scanning electron microscopy (SEM) (Fig 5).

**Figure 6:**
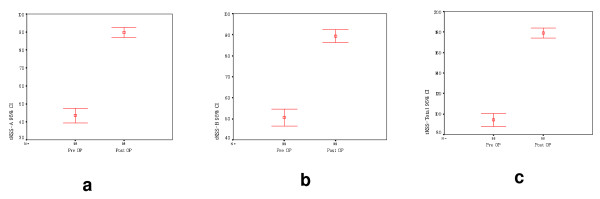
**The error-bar graph illustrating the tKSS-A, tKSS-B, and tKSS-Total scores (95% confidence intervals)**.

Clinician evaluations showed significant mean improvement in the tKSS-A and tKSS-B scores for the all of the data of the three, postoperative, follow-up periods (95% confidence interval, P-value < 0.05) (Fig. 6).

The tKSS-A score showed improvement from 43.52 ± 20.20 to 89.71 ± 13.69 (P < 0.05), while according to the tKSS-B scores, knees were shown to have improved from 50.66 ± 20.05 to 89.38 ± 15.76 (P < 0.05). The total improvement was from 94.18 ± 31.43 to 179.10 ± 24.69 (P < 0.05). Clinical evaluations showed significant mean improvement in the tKSS-A and tKSS-B scores for each postoperative follow-up period (95% confidence interval, P-value < 0.05) (Table 2).

**Table 2:** Overall KSS scores

		Pre-op	Post-op	p-value^1)^
		(Mean ± std)	(Mean ± std)	
tKSS-A		43.52 ± 20.20	89.71 ± 13.69	<0.05
tKSS-B		50.66 ± 20.05	89.38 ± 15.76	<0.05
tKSS-total		94.18 ± 31.43	179.10 ± 24.69	<0.05

Post-op 13~24 months	tKSS-A	47.37 ± 20.56	88.58 ± 15.04	<0.05
	tKSS-B	53.70 ± 18.62	88.70 ± 16.39	<0.05
	tKSS-total	101.08 ± 30.22	177.29 ± 27.19	<0.05
Post-op > 25 months	tKSS-A	37.92 ± 18.53	91.35 ± 11.44	<0.05
	tKSS-B	46.25 ± 21.44	90.37 ± 14.95	<0.05
	tKSS-total	84.17 ± 30.78	181.72 ± 20.57	<0.05

1) Statistical significance was evaluated using the Wilcoxon signed rank test sign test.

According to the follow-up period, improvement of the score of the 'greater than 25 months' group was statistically greater than that of the other group (Table 3). Improvements in tKSS-A and B scores were not found to be related to age, gender, defect size, defect location, the number of vials of chondrocyte used for implantation or the time interval between cartilage harvesting and ACI.

**Table 3:** The tKSS score difference in each group.

		Post-op 13~24 months	Post-op > 25 months	p-value^1)^
			
		(n = 58)	(n = 40)	
tKSS-A Difference	(Mean ± std)	41.20 ± 22.75	53.42 ± 17.95	<0.05
tKSS-B Difference	(Mean ± std)	35.00 ± 20.32	44.12 ± 25.69	<0.05
tKSS-Total Difference	(Mean ± std)	76.20 ± 35.97	97.55 ± 31.41	<0.05

1) Statistical significance was tested using the Mann-Whitney test in each group.

### Adverse events

One patient required repeat surgery five months postoperatively when another cartilage defect was noticed by the surgeon. The amount of improvement in this patient was 40 points total (tKSS-A : 30, tKSS-B : 10) 15 months postoperatively.

Adverse events related to GACI occurred in 2.04% (2/98) of the study patients and were caused by the 'catching symptom'. These 'catching with pain' symptoms subsided postoperatively after they were put on NSAID (Non-Steroidal Anti-Inflammatory Drug) medication.

## Discussion

The surgical procedure of conventional ACI can be summarized as consisting of debridement of the cartilage defect, harvesting of periosteum, suturing the periosteum, and chondrocyte implantation [10]. Among these procedures, periosteum suturing is a difficult and time-consuming procedure for the surgeon, and periosteal harvesting and periosteum suturing procedures have some risk of patient morbidity. Conventional ACI is not preferred because of the periosteal grafting component which requires an additional operation to harvest the periosteum. In addition, for water-tight suturing of the periosteal graft to the surrounding cartilage, a large surgical incision is required, thus presenting the potential problem of subsequent leakage of injected cells from the defect as well as graft detachment [11].

To overcome these potential problems, some researchers have proposed using collagen membrane rather than periosteum [5,11,12]. If the surgeon's preference is to not use periosteum, methods using collagen membrane can eliminate the need for a second incision for periosteal harvest as well as reducing the long surgical time and extensive suturing. However, with this approach there are also some potential problems such as the loss of critical chondrocytes caused by the cutting and repeated manipulation of the seeded membrane. There is also the possibility of detachment of the collagen membrane from the cartilage defect. Therefore, it is becoming increasingly evident that it is necessary to develop a new method.

In the gel-type ACI (GACI), the fibrin can already maintain the shape of the articulation approximately five minutes after injection, thus allowing the cells to remain at the injected sites. And even if there is a defect along the chondral margin, fibrin helps to maintain the shape of the graft according to the articulation [13]. The 5-mm-deep holes serve an important function by increasing the adhesive force of the graft to the defect during knee motion. In the surgical procedures, in order to prevent both the formation of fibrocartilaginous tissue [14] and detachment of the injected cell and fibrin mixture, bleeding control is very important. Fortunately, fibrin sealants are biological adhesives that mimic the final step of the coagulation cascade and therefore help with the bone bleeding control [15]. Also, any commercially available fibrin product can be used because of the similar range of the amounts of fibrinogen and thrombin. Following surgery, we check the stability of the graft by flexion and extension knee-motion exercise. If the graft adequately remains in the defect site, we finish the surgery.

The number of cells introduced in initial explants is important as the seeded chondrocyte number is linearly related to biosynthetic activity [16]. In that respect, GACI can have an advantage over other treatments such as ACI-C and MACI. Collagen membrane occupies quite a substantial amount of space in a cartilage defect, although the cell-gel mixture occupies the space in the GACI. Although the optimal number of required cells has not been determined, high cell densities seem to be desirable [17] and one vial of Chondron™ could cover a total condyle defect. The 96 patients (98.0%) we treated achieved measurable postoperative improvement and only two patients (2.0%) showed deterioration of their 'no change'. One patient required repeat surgery, and all of the patients who underwent surgery showed substantial improvement during the follow-up period. The authors regard these as encouraging results compared with those of other reports regarding marrow stimulation or ACI [18]. In fact, our repeat surgery rate is far less than that associated with other forms of knee surgery [19,20].

However, it is difficult to conclude that this technique is superior to other techniques, such as ACI-C or MACI, as this study is not a comparative study. Many successful results have also been reported using other techniques [6,21,22]. In this study, the authors used a telephone-based scoring system. If the patient does not feel any discomfort, he/she would not need to come to the hospital as job and time limitations frequently prevent patients from checking in with their doctors. Therefore, the necessity and benefits of telephone or internet consultation are increasing. There is also a continuing necessity to develop a new, remote scoring system which can handle the other knee condition evaluations.

Swedish researchers have reported improvements for longer than two years following ACI [23]; similarly, in our study, improvements were more apparent after 24 months. However, the preoperative score of greater than 25-month follow-up group was lower than that of 13~24-month follow-up group. Therefore, it is difficult to insist the same result as Swedish report.

An even more positive aspect of this study is that clinical improvement was apparent even during the short-term, post-op follow-up period of less than two years following surgery.

With respect to the surgical success based on the size of the articular cartilage defects, there was no statistical difference in the results. There was one case of 1 cm^2 ^of cartilage defect and the others were over 2 cm^2^. A small cartilage defect can cause severe discomfort and pain if a patient's activity level is high. The pressure generated during weight-bearing is so high that it stimulates a weak articular cartilage area even though the defect size may be very small. Therefore, covering a cartilage defect with healthy cartilage is very important, and GACI offers a successful treatment option for both small and large cartilage defects.

## Conclusion

GACI used to repair of articular cartilage defects of the knee appears to be a safe and effective method for restoring patient knee function.

## Abbreviations

GACI: gel-type autologous chondrocyte implantation;

## Competing interests

This study was supported by a grant from SewonCellontech, and was followed by the IRB of the Catholic University of Korea. SK is a consultant for SewonCellontech. The other authors declare that they have no competing interests.

## Authors' contributions

SK and NC were involved in collecting patient information, reviewing the literature, study design, and drafting the manuscript as the main authors. BK, WY, HK, JK, YK, YS, JC and CC were involved in the surgery, data collection and study design. DS and HP carried out the ex-vivo evaluation. AA participated in the study design and coordination and helped to draft the manuscript.

All authors read and approved the final manuscript.

## Pre-publication history

The pre-publication history for this paper can be accessed here:

http://www.biomedcentral.com/1471-2474/11/103/prepub
